# Does the Effect of Internet Use on Chinese Citizens’ Psychological Well-Being Differ Based on Their Hukou Category?

**DOI:** 10.3390/ijerph17186680

**Published:** 2020-09-14

**Authors:** Cuihong Long, Jiajun Han, Chengzhi Yi

**Affiliations:** 1 School of Economics, East China Normal University, Shanghai 200062, China; chlong@jjx.ecnu.edu.cn (C.L.); 51194401011@stu.ecnu.edu.cn (J.H.); 2 School of International and Public Affairs, China Institute for Urban Governance, Shanghai Jiaotong University, Shanghai 200030, China

**Keywords:** Chinese hukou system, Internet use, urban–rural gap, psychological well-being, Internet, digital divide

## Abstract

This paper draws support from the 2018 wave of the China Family Panel Studies (CFPS 2018) and uses unconditional quantile regression, re-centered influence function (RIF) decomposition, linear structural equation modelling, extended regression modelling and censored regression to explore the heterogeneity of the impact of Internet use on the psychological well-being of Chinese non-agricultural and agricultural hukou holders. We find that Internet use better improves the psychological well-being of non-agricultural hukou holders, thereby widening the gap in psychological well-being between urban and rural residents in China. Through RIF decomposition, we observe that, except for the 10th quantile, the expansion effect of Internet use on the inequality in psychological well-being between agricultural and non-agricultural hukou holders is mainly reflected in the structure effect, which shows that compared to non-agricultural hukou holders, the return rate of Internet use on the psychological well-being of agricultural hukou holders is lower. Further mechanism analysis shows that using the Internet to socialize, obtain information and understand politics is more beneficial for the psychological well-being of non-agricultural hukou holders; moreover, Internet use can further exert different effects on the psychological well-being of the two groups by differently influencing their job satisfaction, government evaluation, and sleep quality. This study also confirms that relying only on external scientific and technological progress has a limited corrective effect on existing inequalities.

## 1. Introduction

The population of China, the most populous country in the world, is divided into two groups based on the household registration system (commonly known as the hukou system): agricultural hukou holders and non-agricultural hukou holders. In July 1951, the Ministry of Public Security promulgated the “Interim Regulations on the Management of Urban Household Registration”, which stipulated that household registration and management should be implemented in cities to maintain public order while also emphasizing the protection of residents’ freedom of residence and movement. In January 1958, the “Regulations on Household Registration”, passed by the Standing Committee of the National People’s Congress, required citizens moving from rural areas to urban areas to comply with relevant restrictions, which changed the function of the hukou system from population registration and management to population movement control. Each person is assigned a hukou type based on his or her birthplace and lineage, and the social resources that he or she can access, such as education, housing, job opportunities, and health insurance, are tied to his or her hukou category. Since 1978, with the initiation and continuous advancement of the reform and opening up, the hukou system has gradually loosened, but its role has not fundamentally changed. The hukou system is considered an important means of allocating public resources in China, and it has brought profound social, economic and political consequences, including the urban–rural gap in social development, income disparities and inequality of rights [1,2]. Thus, this system often places agricultural hukou holders at a disadvantage in terms of access to resources such as education, housing, and social security [1,2]. Many scholars have noted that the differences in social welfare hidden behind the hukou system have an adverse effect on the subjective well-being, physical health, and psychological well-being of agricultural hukou holders [3,4,5,6,7,8], and they have provided helpful insights into understanding the differences in subjective well-being between Chinese agricultural and non-agricultural hukou holders.

Exploring the relationship between Internet use and the psychological well-being of Chinese agricultural and non-agricultural hukou holders is particularly important. In recent years, the Internet has developed rapidly in China. As of June 2019, the number of Chinese Internet users had reached 854 million [9], constituting the largest group of netizens in the world. Internet expansion has had a wide range of impacts on the lifestyle, employment, and physical and psychological health of Chinese residents. Some scholars have noted that the Internet has played an important role in improving users’ physical health, psychological well-being, life satisfaction and subjective well-being [10,11] and in alleviating their loneliness and depression [12,13]. Castellacci and Tveito summarized that Internet use can impact the well-being of its users through the four channels of changing time use patterns, creating new activities, facilitating access to information and acting as a powerful communication tool [14]. However, affected by China’s long-term urban–rural dual structure, the development of the Internet has also led to a digital divide between urban and rural areas [15,16,17]. Zhang proposed two theories pertaining to the urban–rural digital gap from the perspective of the impact of the Internet on urban–rural educational equity: (1) gap narrowing theory, which proposes that the expansion of the Internet has brought high-quality educational resources to rural students that help narrow the gap in urban–rural education and promote educational equity, which in turn narrows the urban–rural gap formed by the hukou system; and (2) gap strengthening theory, which suggests that the information capital generated by the Internet will continuously strengthen existing social inequality through mutual transformation with other forms of capital [18]. We believe that, on the one hand, as a new means of resource allocation, the Internet can offer new opportunities for agricultural hukou holders. For instance, rural residents can use the Internet to sell agricultural products and obtain employment information, and the Internet can be used to address livelihood issues through government websites. Consequently, using the Internet can narrow the gap between agricultural and non-agricultural hukou holders. On the other hand, the social stratification resulting from the hukou system has placed urban residents ahead of rural residents in many ways. The ability to use the Internet and preferences for Internet use may vary greatly between these two groups. Non-agricultural hukou holders may make more effective use of the Internet to consolidate their “information-rich” status, while agricultural hukou holders may be unable to quickly acquire digital skills and break through the “information-poor” class ceiling. Thus, the possession and use of information capital will become a new mechanism for maintaining or even expanding the reproduction of inequality between urban and rural areas [17]. Internet use may have different effects on improving the psychological well-being of urban and rural residents in China [10,11]. However, whether and how Internet use affects the psychological well-being of Chinese urban and rural residents has not been sufficiently explored in the existing literature. In this context, exploring the impact of Internet use on the psychological well-being of Chinese urban and rural residents holds important practical significance.

Observing intrahousehold resource allocation in the United States, Frijters et al. found that parents have an investment preference for high-ability children; that is, children with stronger initial cognitive ability will obtain more resources from their parents. Therefore, the ability gap between children is solidified by differential parental resource allocation, with strong children becoming stronger and weak children becoming weaker [19]. As mentioned above, China’s hukou system plays an important role in resource allocation, and it makes the psychological well-being of agricultural hukou holders weaker than that of non-agricultural hukou holders. Will the popularization of the Internet help break the long-term unbalanced model of resource allocation between urban and rural areas and decrease the gap in psychological well-being between urban and rural residents? Alternatively, will the popularization of the Internet better improve the psychological well-being of non-agricultural hukou holders and further widen the gap in psychological well-being between urban and rural residents? If so, through what channels will this effect occur? The answers to these questions will help clarify the impact of the Internet on the psychological well-being of Chinese urban and rural residents and the mechanisms involved. Furthermore, they will provide new ideas for improving the psychological well-being of residents in other developing countries.

To enrich research on the impact of the Internet on the psychological well-being of urban and rural residents in China, we use data from the 2018 wave of the China Family Panel Studies (CFPS 2018) and comprehensively apply unconditional quantile regression, re-centered influence function (RIF) decomposition, linear structural equation modelling, extended regression modelling and censored regression to examine the differential impact of Internet use on the psychological well-being of urban and rural residents in China.

## 2. Materials and Methods

### 2.1. Literature Review

With the rapid development of the Internet, an increasing number of scholars have revealed the impact of the Internet on the psychological well-being of users. Bessière et al. [20] proposed three hypotheses regarding the effect of the Internet on psychological well-being from the perspective of social resource allocation: (1) the social augmentation hypothesis, which suggests that communication on the Internet can improve psychological well-being by offering additional avenues of social interaction and enlarging the user’s social networks; (2) the social displacement hypothesis, which proposes that online communication displaces valuable routine social interaction with family and friends and negatively impacts the psychological well-being of users; and (3) the social compensation hypothesis, which holds that Internet users who have initially impoverished social resources can meet new friends and participate in new groups through the Internet, with online activities thus being able to help compensate for the social resources such users lack in the offline world. We observe that, on the one hand, compared with urban residents, residents of China’s rural areas belong to an “acquaintance society” and that rural residents communicate with their family and friends more frequently than urban residents. From this perspective, the Internet may be more conducive to enhancing the psychological well-being of non-agricultural hukou users. On the other hand, the hukou system has dominated the allocation of resources in mainland China since the 1950s. Liu found that agricultural hukou holders often have fewer educational resources, lower rates of return on education, and limited employment choice in inflow cities [1]. Song and Smith suggested that the health inequalities between non-agricultural and agricultural hukou holders can be attributed to the differences in medical services and family backgrounds since childhood and the lower number of clean water resources in rural areas than in urban areas [5]. In the past decade, the resource allocation effect exerted by the Internet has likely interacted with the hukou system and had different effects on the psychological well-being of non-agricultural and agricultural hukou holders. This assertion has been supported by the findings of some researchers. Zhou and Sun found that an improvement in the subjective well-being of Internet users mainly occurred among residents in large and medium-sized cities [10]. Wang found that Internet use can significantly improve the psychological well-being of elderly urban residents in China but has no significant impact on the psychological well-being of elderly rural residents [11].

The Internet is thought to form a digital divide between urban and rural areas. Cheng et al. noted that the digital gap among provinces in China is significant and that the network competitiveness of the eastern coastal areas is generally ahead of that of China’s central and western regions, especially regarding the network infrastructure in the western region, which is less developed; thus, network competitiveness is facing a low-end lock-in dilemma [21]. However, with the rapid increase in the Internet penetration rate in recent years, Qiu et al. indicated that the difference in Internet accessibility between urban and rural areas is gradually decreasing and that the digital gap between urban and rural areas is instead more reflected in urban and rural users’ unequal ability to draw benefits from Internet use [22]. Tan et al. emphasized the difference in the return rate between urban and rural residents using the Internet in China and attributed the digital gap between urban and rural areas to agricultural hukou holders’ lack of ability to apply and appreciate Internet use [23]. Some scholars have also noted that rural students use the Internet more for entertainment, while urban students use the Internet more for information [16,24]. Accordingly, the first hypothesis is as follows.

**Hypothesis** **1.** *Internet use can better improve the psychological well-being of non-agricultural hukou holders compared to the psychological well-being of agricultural hukou holders, which is mainly reflected in the higher rate of return of non-agricultural hukou holders’ Internet use on their psychological well-being.*

If the Internet is more conducive to improving the psychological well-being of non-agricultural hukou users compared to agricultural hukou users, then through what channels is this heterogeneity formed? Judge and Bono found that an improvement in job satisfaction could significantly improve workers’ self-esteem, self-efficacy, and emotional stability, thereby improving their psychological well-being [25]. Martin and Omrani reported that Internet use was positively related to employees’ job satisfaction [26]. More scholars have explored the mechanism through which Internet use affects job satisfaction. Hua et al. showed that the use of the Internet by taxi drivers in the era of the sharing economy could help reduce their work pressure and increase their work autonomy, thereby improving their job satisfaction [27]. However, Wang et al. indicated that increased Internet use led to greater perceptions of family-to-work conflict, which further decreased job satisfaction [28]. Castellacci and Viñas-Bardolet found that the Internet could enhance users’ job satisfaction in three ways, i.e., promoting access to online information, creating new activities, and improving interpersonal communication, but only for white-collar workers; on the other hand, Internet use actually led to lower job satisfaction for blue-collar workers [29].

In addition to job satisfaction, the relationship between social capital and psychological well-being has received scholarly attention. Social capital is a concept that has received long-term attention and extensive discussion in the social sciences. According to Putnam [30], its core elements focus on features including social trust, networks and norms. Social capital has been classified into three different types, that is, bonding, bridging and linking social capital, in which bonding and bridging social capital emphasize horizontal ties, while linking social capital emphasizes vertical ties [31]. Huang and Liu suggested that bonding social capital online mediated the relationship between information use and job satisfaction [32]. Some studies have revealed a significant positive relationship between psychological well-being and linking social capital, measured by political trust, evaluations of political effectiveness and political participation [33,34]. The Internet plays various roles politically, and it has become an important channel for citizens to access government information, express different opinions, engage in public participation, and supervise the government. Existing research shows that Internet use also has a complex impact on government evaluation. Hong suggested that online experiences were positively associated with government trust at the local and state levels [35]. Ceron found that obtaining political information from information websites enhanced citizens’ trust in the government; however, obtaining information through social media reduced their trust in the government [36]. Chen reported that citizens who used the Internet tended to trust the central government less than those who did not [37]. Lu et al. suggested that Internet use was significantly positively related to netizens’ satisfaction with government performance but negatively associated with deference to government authority [38]. In another vein, studies have shown that a decline in sleep quality has a significant positive correlation with an increase in depression and a decrease in psychological well-being [39,40,41]. Excessive Internet use has also been found to have a significant positive correlation with insufficient sleep and decreased sleep quality [42,43,44]. Ning et al. found that Internet use significantly reduced the sleep time of Chinese rural teenagers [45]. Given that many of the economic and social resources of Chinese urban and rural residents are closely related to their hukou status and given that very large gaps in Internet use time and preferences exist between non-agricultural and agricultural hukou holders, we propose the second hypothesis.

**Hypothesis** **2.** *Internet use can have different effects on the psychological well-being of non-agricultural and agricultural hukou holders by differently influencing their job satisfaction, government evaluation, and sleep quality.*

### 2.2. Data, Variables and Methods

#### 2.2.1. Data Source and Variables

The data used in this paper are from CFPS 2018. The CFPS is conducted by the Institute of Social Science Survey (ISSS) of Peking University and covers 25 provinces/cities/autonomous regions with a sample size of 16,000 households. The CFPS is nationally representative, and the results obtained from our empirical results can be extrapolated to the entire Chinese population. For the CFPS, computer-assisted personal interviewing (CAPI) is conducted with all family members in the sample households to improve access efficiency and ensure data quality. The CFPS includes the administration of a questionnaire about Internet use among Chinese citizens that collects data on whether respondents use the Internet and specific Internet usage preference variables, such as the frequency of socializing via the Internet, the importance of using the Internet to obtain information, and the frequency of learning about politics via the Internet. Data on the personal characteristics, mental state and subjective attitudes of Chinese citizens, which are appropriate for our research, are also collected in the CFPS. The main research variables selected are shown in Table 1.

**Figure 1:**
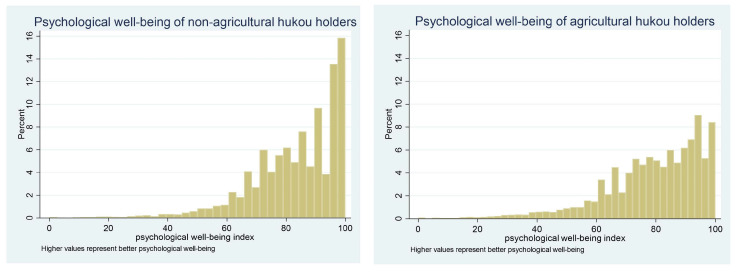
Distribution of the psychological well-being index among non-agricultural and agricultural hukou holders.

As mentioned above, there are wide gaps in many aspects of urban and rural China due to the hukou system. Before employing the empirical strategy, we graphically illustrate the distribution of the psychological well-being index of agricultural hukou holders and non-agricultural hukou holders. We select seven specific questions from the Center for Epidemiologic Studies Depression (CESD) scale used in CFPS 2018, including questions on the frequency of the perception of depression, stress, happiness, loneliness, enjoyment of life, sadness, and desperation in the past week (anchored by 1 = less than one day and 4 = 5–7 days) to construct the factor of psychological well-being (Cronbach’s α = 0.7712, overall KMO = 0.7774). The principal factor method using iterated communalities is employed here. According to Hamilton [46], iterated principal factors will indicate the latent dimensions that best reveal the correlation patterns among variables. After the factor is rotated, we convert this factor into a continuous comprehensive index of respondents’ psychological well-being that ranges from 0 to 100 [47], with a higher value indicating better psychological well-being. As shown in Figure 1, the psychological well-being among non-agricultural hukou holders is better than that among agricultural hukou holders.

Figure 1 shows that compared to non-agricultural hukou holders, agricultural hukou holders are always at a disadvantage in terms of their psychological well-being. In the next part, we explore whether the Internet can exacerbate this disparity by further improving the psychological well-being of non-agricultural hukou holders or alleviate this inequality by better ameliorating the psychological well-being of agricultural hukou holders. Owing to the many Internet use-related variables measured in CFPS 2018, we can observe the effect of specific Internet use methods, which sheds some light on the mechanism of this effect.

#### 2.2.2. Methods

This article uses unconditional quantile regression, RIF decomposition, linear structural equation modelling, extended regression modelling and censored regression to examine the heterogeneity of the impact of Internet use on the psychological well-being of Chinese non-agricultural and agricultural hukou holders.

## 3. Results

We select the previously generated psychological well-being index as our dependent variable and draw support from RIF regression to employ unconditional quantile regression, which is a regression framework analysis technique for exploring the factors underlying changes across unconditional distributions. Compared to conditional quantile regression, unconditional quantile regression offers a direct, concise way of estimating the effect of independent variables at all points of the distribution of the dependent variable [48]. In this article, the unconditional quantile treatment effects estimate the effect of a treatment (i.e., Internet use) on the marginal psychological well-being distribution of the entire population. In Equation (1), “*psy_well_i_*” represents the psychological well-being of the *i*-th respondent, and RIF(*psy_well_i_*, *v*(*F_Psy_well_*)) is the contribution of a *psy_well_i_* observation on the construction of statistic *v*, where *v* represents the functional of the cumulative distribution function of the dependent variable. For the τth quantile, the influence function (IF) is equal to (*τ* − 1(*Psy_well* ≤ *qτ*))/*f_Psy_well_*(*qτ*). 1(.) is an indicator function indicating whether the value of the outcome variable is below *qτ*, and *f_Psy_well_* is the probability density function of our dependent variable. RIF(*psy_well_i_*, *qτ*, *F_Psy_well_*) is equal to *qτ* + (*τ* − 1(*Psy_well* ≤ *qτ*))/*f_Psy_well_*(*qτ*). In the case of the mean, the RIF is simply the independent variable itself, and the corresponding RIF regression is equivalent to ordinary least squares (OLS) regression. “*Internet_i_*” represents whether the *i*-th respondent uses the Internet, which is our critical independent variable; “*controls_i_*” represents the control variables; and “*ε_i_*” represents the error term. We choose the 10th quantile, 25th quantile, mean, 75th quantile, and 90th quantile to define the distributional statistic to be used for the RIF regression.

(1) $$\mathrm{RIF}\left(psy\_well_{i},v\left(F_{Psy\_well}\right)\right)=\alpha +\beta Internet_{i}+\phi control_{i}+\varepsilon_{i}^{}$$

We select 10 control variables: age, gender, marital status, and years of education, which serve as demographic variables; income level, life satisfaction, social status, and Chinese Communist Party (CCP) member status, which serve as socioeconomic variables; and smoking status and frequency of alcohol consumption, which serve as health-related behavioral variables. In the RIF regression, we identify the fixed effect of the province to be absorbed. Here, we use the STATA command “rifhdreg” to conduct the RIF regression and select the option “absorb(province)” to identify the fixed effect of the province to be absorbed. As shown in Table 2 (where non-agricultural hukou holders are labelled “urban” and agricultural hukou holders are labelled “rural”, which is similarly applied in the subsequent tables), the coefficients of the internet variable in the non-agricultural group are greater and more significant than those in the agricultural group, regardless of the quantile we choose, especially at the lower end of the psychological well-being distribution. In columns (1), (3) and (5), the coefficients of internet are all positive and significant at the 1%, 10%, and 5% levels, respectively, while in columns (2), (4) and (6), the absolute values of the coefficients of internet all decrease and become nonsignificant in columns (4) and (6), which suggests that Internet use can better improve psychological well-being for non-agricultural hukou holders than for agricultural hukou holders. Regarding the control variables, the coefficients of the variable female are all negative and significant at the 1‰ level, suggesting that Chinese women always have worse psychological well-being than their male counterparts. The coefficients of the variables age and single are lower for agricultural hukou holders, which demonstrates that to a certain extent urbanization can buffer the negative effects of ageing and being single on psychological well-being. Interestingly, the coefficients of the variable years of education are higher for agricultural hukou holders, which suggests that being better educated can improve psychological well-being more for agricultural hukou holders than for non-agricultural hukou holders in China.

Since Internet use can better improve the psychological well-being of non-agricultural hukou holders and since Figure 1 shows that their psychological well-being is always better than that of agricultural hukou holders, we next perform RIF decomposition to decompose the inequality of the same statistics chosen in Table 2, analyzing the differences by hukou category.

(2) $$\begin{array}{ll} \Delta v & =\left(v_{\mathrm{non}-\mathrm{agri}}-v_{\mathrm{counterfactual}}\right)+\left(v_{\mathrm{counterfactual}}-v_{\mathrm{agri}}\right)\\ & =[v\left(\int F_{Psy\_well|X,\mathrm{non}-\mathrm{agri}=1}dF_{X|\mathrm{non}-\mathrm{agri}=1}\right)-v\left(\int F_{Psy\_well|X,\mathrm{agri}=1}dF_{X|\mathrm{non}-\mathrm{agri}=1}\right)]+\\ & \;[v\left(\int F_{Psy\_well|X,\mathrm{agri}=1}dF_{X|\mathrm{non}-\mathrm{agri}=1}\right)-v\left(\int F_{Psy\_well|X,\mathrm{agri}=1}dF_{X|\mathrm{agri}=1}\right)]\\ & =\Delta_{\mathrm{structure}}^{v}+\Delta_{\mathrm{composition}}^{v} \end{array}$$

In Equation (2), *v* represents the functional of the cumulative distribution function of the dependent variable, i.e., psychological well-being; $\Delta_{\mathrm{composition}}^{v}$ reflects the effect of differences in the distribution of independent variables, which represents the composition effect; and $\Delta_{\mathrm{structure}}^{v}$ reflects changes in the psychological well-being structure function, which represents the structure effect. In other words, these differences are featured by functions of differences in characteristics (composition effect) and the differences in the coefficients connected with those characteristics (structure effect) [49]. As shown in Table 3, the sum of the explained and unexplained parts of the coefficients of the variable internet are all positive for every quantile we choose, which suggests that Internet use can further widen the gap in psychological well-being between non-agricultural and agricultural hukou holders. In column (2), the explained part of the coefficient of internet is positive and significant at the 5% level, and the unexplained part of the coefficient is nonsignificant, suggesting a widened gap in psychological well-being caused by the different Internet usage rates between the two groups of hukou holders in the 10th percentile. In columns (6), (9) and (15), as the quantile increases, the unexplained parts of the coefficients of internet are all positive and significant at the 10%, 5%, and 10% levels, respectively. The explained parts of the coefficients become nonsignificant, which suggests that Internet use may intensify the disparity in psychological well-being through the structure effect, i.e., widening the gap in psychological well-being. Different return rates of Internet use on psychological well-being between non-agricultural and agricultural hukou holders exacerbate the inequality of psychological well-being between these two groups. This finding is consistent with existing research; the digital gap between rural and urban China (both the gap in the penetration rate and the difference in use preferences) can be unfavorable for agricultural hukou holders and tilt the balance in favor of non-agricultural hukou holders [16,24,50]. Thus, introducing the Internet cannot redress the inequality in psychological well-being between non-agricultural and agricultural hukou holders, and we observe agricultural hukou holders’ disadvantage in the psychological well-being distribution, with most of the disadvantage being driven by the structure effect.

We verify that Internet use can better improve non-agricultural hukou holders’ psychological well-being compared to agricultural holders’ psychological well-being in China and, more interestingly, that the potential channels through which Internet use affects users’ psychological well-being differ by hukou category. First, we transform our critical explanatory variable, i.e., Internet use, into the specific Internet use method or preference, while the dependent variable and other control variables remain the same.

(3) $${psy\_well}_{i}=\alpha +\beta {Internet\mathrm{mod}e}_{i}+\phi {controls}_{i}+\varepsilon_{i}$$

We employ linear regression, absorbing multiple levels of fixed effects to explore the mechanism and identify the provincial fixed effects to be absorbed. Here, we use the STATA command “reghdfe” to conduct the mechanism analysis and select the option “absorb(province)” to identify the fixed effect of the province to be absorbed. In Equation (3), we select three different Internet use-related variables: the frequency of socializing via the Internet, the importance of using the Internet to obtain information, and the frequency of learning about politics via the Internet. As shown in Table 4, column (1), the coefficient of the variable frequency of socializing via the Internet is positive and significant at the 5% level. In column (2), the coefficient is nonsignificant, suggesting that socializing via the Internet can better improve the psychological well-being of non-agricultural hukou holders. In column (3), the coefficient of the variable importance of using the Internet to obtain information is nonsignificant. In column (4), this coefficient is negative and significant at the 1‰ level, which suggests that giving more weight to the role of the Internet in providing information can decrease the psychological well-being of agricultural hukou holders. In column (5), the coefficient of the variable learning about politics via the Internet is positive and significant at the 5% level. In column (6), the coefficient is nonsignificant, suggesting that access to political information via the Internet can better improve the psychological well-being of non-agricultural hukou holders. Therefore, Internet use can be more beneficial for non-agricultural hukou holders, further enlarging the gap in psychological well-being between the two categories of hukou holders.

Second, we once again select Internet use as our critical independent variable and select job satisfaction, government evaluation, and frequency of poor sleep as our intervening variables to construct a linear structural equation model (the details of the intervening variables are shown in Table 1).

(4) $${psy\_well}_{i}=\alpha_{1}+\theta_{}{mechanism}_{i}+\beta_{1}{Internet}_{i}+\phi_{1}{controls}_{i}+\varepsilon_{i1}$$

(5) $${mechanism}_{i}=\alpha_{2}+\beta_{2}{Internet}_{i}+\phi_{2}{controls}_{i}+\varepsilon_{i2}$$

In Equations (4) and (5), *mechanism_i_* represents an intervening variable. Luo indicated a positive association between employment and happiness [51]. Castellacci and Viñas-Bardolet found that Internet use improved only white-collar workers’ job satisfaction and decreased the job satisfaction of blue-collar workers [34]. Tan et al. reported that the Internet could increase working income for Chinese urban citizens; however, there was no significant relationship between Internet use and working income for Chinese rural residents [23]. We select job satisfaction as an intervening variable to explore the potential mechanism. As shown in Table 5, columns (1) and (3), the coefficients of job satisfaction are both positive and significant at the 1‰ level, indicating a significant association between job satisfaction and psychological well-being for both non-agricultural and agricultural hukou holders. In column (2), the coefficient of Internet use is positive and significant at the 10% level, suggesting that it can increase job satisfaction among non-agricultural hukou holders and improve their psychological well-being; however, in column (4), the coefficient of Internet use is negative and significant at the 10% level, suggesting that it can decrease the job satisfaction of agricultural hukou holders and further decrease their psychological well-being. As a result, Internet use can better improve non-agricultural hukou holders’ psychological well-being. Chen et al. suggested that occupational segregation due to the hukou barrier is attributable to the depressive effect of the hukou system on agricultural hukou holders’ well-being, arguing that the demonstration effect overtakes the comparison effect for non-agricultural hukou holders but not for agricultural hukou holders [52]. We also suggest that the demonstration effect of Internet use outstrips the comparison effect for non-agricultural hukou holders; without the hukou threshold, they could better utilize the Internet. In contrast, the comparison effect may be greater than the demonstration effect for agricultural hukou holders, and their job satisfaction might be negatively associated with Internet use. Ultimately, the Internet can affect psychological well-being differently based on the hukou category of users by influencing their job satisfaction.

Jiang and Wang identified political trust, political participation and political efficacy as forms of linking social capital and discovered a significant positive association between linking social capital and psychological health [34]. We also select government evaluation as an intervening variable. As shown in Table 5, in columns (5) and (7), the coefficients of government evaluation are both positive and significant at the 1‰ level, indicating a significant positive association between government evaluation and the psychological well-being of both non-agricultural and agricultural hukou holders. In column (6), the coefficient of Internet use is positive and significant at the 1% level, suggesting that it can improve non-agricultural hukou holders’ evaluation of the government and further improve their psychological well-being. In contrast, in column (8), the coefficient of Internet use is negative and significant at the 1‰ level, which suggests that it can have a depressive effect on agricultural hukou holders’ evaluation of the government; thus, Internet use can better improve non-agricultural hukou holders’ psychological well-being. Hong found that a successful experience with Internet channels could be more important than the experience itself in terms of government trust [35]. On the one hand, Internet devices are always worse in rural China than in urban China; on the other hand, news pertaining to rural migrant workers facing wage arrears and discrimination is very common in Chinese media, and agricultural hukou holders may have unsuccessful experiences more frequently than non-agricultural hukou holders. Considering the unequal resource allocation generated by the hukou system, agricultural hukou holders may suffer unfair treatment more frequently than their urban counterparts. Hence, the Internet can have different effects on users’ psychological well-being beyond their hukou category by differently influencing their evaluation of the government.

**Figure 2:**
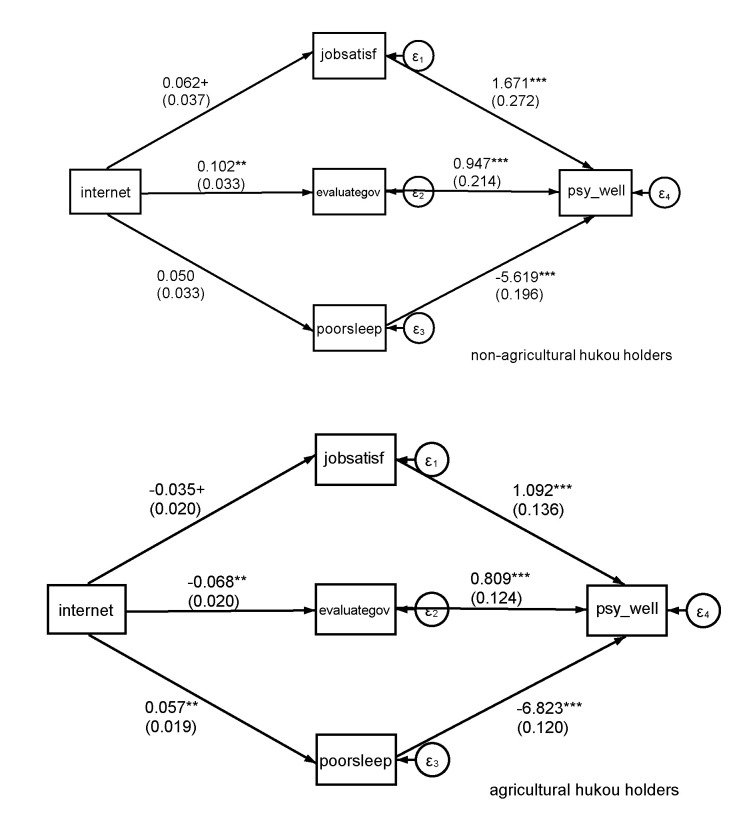
Graphical representation of the linear structural equation model in Table 5 (including the control). *** *p* < 0.001, ** *p* < 0.01, + *p* < 0.1.

Woods and Scott revealed a positive association of intensive social media use and poor sleep, lower self-esteem, anxiety and depression [53]. Viner et al. attributed the mental harm related to intensive social media use to a reduction in sleep or physical exercise [41]. Hisler et al. indicated that surfing the Internet could lead to multiple dimensions of impaired sleep [44]. We also select the frequency of poor sleep as an intervening variable. As shown in Table 5, in columns (9) and (11), the coefficients of poor sleep are both negative and significant at the 1‰ level, which suggests that poor sleep can damage the psychological well-being of both non-agricultural and agricultural hukou holders. In column (10), the coefficient of Internet use is nonsignificant, which suggests that it may not impair the sleep quality of non-agricultural hukou holders. By comparison, in column (12), the coefficient of Internet use is positive and significant at the 1% level, suggesting that it can increase the likelihood of poor sleep and further weaken the psychological well-being of agricultural hukou holders. Consequently, Internet use can tilt the balance in favor of non-agricultural hukou holders’ psychological well-being. Meng and Zhang [54] noted that agricultural hukou holders are always concentrated in physically demanding jobs. In contrast, non-agricultural hukou holders are more likely to be white-collar workers; thus, their adaptability to the Internet environment may be different, and Internet use is more likely to impair agricultural hukou holders’ sleep quality. Ultimately, the Internet can have different impacts on users’ psychological well-being based on their hukou category by differently influencing their sleep quality. Besides Table 5, we also illustrate the results of linear structural equation model more intuitively in the form of diagram in Figure 2.

## 4. Discussion

Our empirical results effectively corroborate the two hypotheses. As mentioned above, the hukou system has dominated resource allocation for approximately seven decades, placing agricultural hukou holders at a disadvantage. Meanwhile, as an advanced technology, the Internet has failed to change the long-term inequalities between urban and rural residents in China, but better improved non-agricultural hukou holders’ psychological well-being. The results of unconditional quantile regression show that the Internet can better improve the psychological well-being of non-agricultural hukou users, and this improvement is especially significant when the quantiles selected are lower, which suggests that non-agricultural hukou holders with worse initial psychological well-being can better improve their condition by using the Internet compared to their agricultural counterparts in the same situation. When we change the critical independent variable from Internet use to the specific Internet usage method, the results still support Hypothesis 1. We employ RIF decomposition to split the psychological effect of Internet use based on the disparity between agricultural and non-agricultural hukou holders, which are the composition effect (differences in the statistics of interest ∆*v* will arise because of the differences in the distribution of the independent variable) and the structure effect (because of the differences in the relationships between the dependent variable and independent variable). Our results reveal that the widening effect of Internet use on the disparity in psychological well-being between agricultural and non-agricultural hukou holders is mainly reflected in the structure effect and that agricultural hukou holders’ poor ability to use the Internet is the primary reason the Internet tilts the balance in favor of non-agricultural users. The results not only echo those of existing research [10,11,21] and confirm Hypothesis 1 but also demonstrate that agricultural hukou holders should improve their ability to use the Internet effectively since the role of the hukou system in signaling permission to access public resources has been entrenched for over half a century. Thus, simply introducing a new technology such as the Internet may not benefit the initially disadvantaged groups, i.e., agricultural hukou holders.

The results of our linear structural equation model substantiate Hypothesis 2: Internet use can better improve the job satisfaction and government evaluation of non-agricultural hukou holders, and it can worsen sleep quality more for agricultural hukou holders than for non-agricultural hukou holders. Therefore, the Internet may tilt the balance away from agricultural users, with non-agricultural hukou holders standing to benefit more from using the Internet, based on previous studies [29,37,45]. The confirmation of Hypothesis 2 implies that the government can enhance the linkage between the Internet and agricultural hukou holders’ jobs, especially in the context of the sharing economy. Authorities should further loosen the hukou system. Once Chinese agricultural migrant workers are able to fairly work and access public resources in the inflow city and their children who would otherwise be left behind are able to equally attend urban schools, they could more easily choose to bring their families with them and family companionship should be able to weaken such workers’ dependence on the Internet; consequently, their sleep quality may be less affected by the Internet.

In the next part, we use linear regression, absorbing multiple levels of fixed effects, to analyze the heterogeneity of Internet use for non-agricultural and agricultural hukou holders, and we also identify the fixed effect of the province to be absorbed. Here, we use the STATA command “reghdfe” to conduct the heterogeneity analysis and select the option “absorb(province)” to identify the fixed effect of the province to be absorbed. We construct the interaction term combining Internet use with gender, age, marital status, years of education, and income level. As shown in Table 6, column (1), the coefficient of the interaction term combining the variables internet and female is positive and significant at the 1% level, suggesting that Internet use can better improve female users’ psychological well-being compared to male users’ well-being among non-agricultural hukou holders. However, in column (2), the coefficient of the interaction term is nonsignificant, suggesting that there is no heterogeneity in the psychological effect between genders among agricultural hukou holders. In columns (3) and (4), the coefficients of the interaction term combining the variables internet and age are both positive and significant at the 5% level, which suggests that Internet use can improve the psychological well-being of elderly users in both the non-agricultural and agricultural hukou groups. In columns (5) and (6), the coefficients of the interaction term combining the variables internet and single are both positive and significant at the 1‰ level, which suggests that single people can benefit more from using the Internet than people with a different marital status. In columns (7) and (8), the coefficients of the interaction term combining the variables internet and years of education are both negative and significant at the 5% level, suggesting that Internet use may compensate for the effect of a lack of education on psychological well-being for both non-agricultural and agricultural hukou holders. In columns (9) and (10), the coefficients of the interaction term combining the variables internet and income level are both nonsignificant, which demonstrates that there is no heterogeneity in the effect of Internet use on psychological well-being based on the income level of both non-agricultural and agricultural hukou holders.

Next, we run the extended regression model to address any potential concerns about endogeneity. It can be argued that respondents who have lower psychological well-being are less likely to talk to others offline and therefore prefer to obtain information online or that they are more likely to seek solace or approval through the Internet or regard the Internet as a means of catharsis. In contrast, those whose psychological well-being is higher may want to share their lifestyles online more often. If this assumption holds, it could trigger a bidirectional causality problem. Chen noted that the preference for ICT devices such as mobile phones and computers should reflect a kind of consumption heterogeneity at the personal level and not have a direct association with personal well-being [55]. We select the use of a cell phone as an instrumental variable and employ the extended regression model to allay any potential concerns about endogeneity. Not only does the extended regression model allow more flexibility regarding the form of the dependent variable and endogenous variable, such as continuous, binary, ordered, or truncated, but it also allows endogenous covariates in interactions, whereas early commands in STATA such as ivregress or ivprobit did not allow this condition [34,56].

(6) $${psy\_well}_{i}=\lambda_{0}+\lambda_{1}{Internet}_{i}+\lambda_{2}{controls}_{i}+{e.}_{}{psy\_well}_{i}$$

(7) $${Internet}_{i}=\eta_{0}+\eta_{1}{IV}_{i}+\eta_{2}{{controls}_{i}+e.}_{}{Internet}_{i}$$

The main model is Equation (6), and the endogeneity model is Equation (7). *IV_i_* represents the instrumental variable, i.e., the use of a cell phone, and *e.psy_well_i_* and *e.Internet_i_* denote the residuals of psychological well-being and Internet use, respectively. As shown in Table 7, columns (2) and (4), the coefficients of the variable cellphone are both positive and significant at the 1‰ level, suggesting a robust correlation between the use of the Internet and the use of a cell phone, excluding the possibility of weak instrumental variables. In column (1), the coefficient of the variable internet is positive and significant at the 5% level for non-agricultural hukou holders. However, in column (3), the coefficient of internet is nonsignificant, and the absolute value is drastically decreased, which suggests that Internet use can better improve the psychological well-being of non-agricultural hukou holders.

Notably, Corr(e.psy_well, e.internet) in Table 7 is nonsignificant for both the non-agricultural and agricultural groups, which shows that the residuals between psychological well-being and Internet use are barely correlated, suggesting that there are negligible endogeneity problems in the early regressions and that our initial empirical results are robust.

In the last part, we conduct the robustness test; considering the distribution of the psychological well-being index in Figure 1, we carry out censored regression with the upper limit and regard the maximum value of 100 as the upper limit. As shown in Table 8, column (1), the coefficient of Internet use is positive and significant at the 1% level for non-agricultural hukou holders. By comparison, in column (2), the coefficient is nonsignificant, and its absolute value is drastically lower in the agricultural group than in the non-agricultural group, suggesting that Internet use can better improve non-agricultural hukou holders’ psychological well-being. When we transform the critical independent variable into the frequency of Internet use to socialize and learn about politics, we observe the same scenario in columns (3), (4), (7) and (8). In column (5), the coefficient of the importance of using the Internet to obtain information is nonsignificant for non-agricultural hukou holders, while in column (6), this coefficient is negative and significant at the 1‰ level, suggesting a depressive effect of accessing information via the Internet on agricultural hukou holders’ psychological well-being. Ultimately, Internet use will work to the advantage of non-agricultural hukou holders.

## 5. Conclusions

Both the hukou system and the Internet have the function of resource allocation, and they are two important means of resource allocation. China has become the country with the largest number of Internet users in the world. However, there is a digital divide between urban and rural areas. The research concerning the heterogeneity of the impact of the Internet on the psychological well-being of users based on their hukou category lacks sufficient attention and detailed discussion. Based on data from CFPS 2018, we used unconditional quantile regression and RIF decomposition to investigate the heterogeneity of the impact of Internet use on the psychological well-being of non-agricultural and agricultural hukou holders. We also employed linear regression, absorbing multiple levels of fixed effects, and a linear structural equation model to explore the impact mechanism. Furthermore, we performed censored regression to test the robustness of our results. Our empirical results show that the Internet will be more conducive to improving the psychological well-being of non-agricultural hukou users, thus widening the gap in psychological well-being between urban and rural residents in China. The RIF decomposition indicates that the expansion of this gap is mainly reflected in the structure effect, suggesting that with the convergence of urban and rural Internet penetration rates, the urban–rural digital divide is more reflected in the difference in the return rates on Internet use for non-agricultural and agricultural hukou holders. This difference also shows that the ability of agricultural hukou holders to use the Internet needs to be improved. Conducting mechanism analysis, we found that using the Internet to socialize, obtain information and learn about politics can improve the psychological well-being of non-agricultural hukou holders. Moreover, Internet use can exert different effects on job satisfaction, government evaluation and sleep quality between non-agricultural and agricultural hukou holders, in turn having different effects on their psychological well-being. The heterogeneity analysis indicated that the Internet is more conducive to improving the psychological well-being of older people, single people, and people with fewer years of education.

Our research also has important implications for future government policy. Since the hukou system has been a determining factor in resource allocation for more than half a century in mainland China, compared to non-agricultural hukou holders, agricultural hukou holders have been at a disadvantage in terms of their psychological well-being. We find that as a newly developed technology, the Internet can also allocate resources; however, agricultural hukou holders lack the ability to make use of the Internet, and non-agricultural hukou holders can better benefit from using the Internet. Possibility always requires permission, and Chinese authorities should continuously relax the hukou system, grant more social resources to agricultural hukou holders, and cultivate agricultural hukou holders’ ability to effectively use new resources. Although restrictions on migration have gradually disappeared, rural migrant workers are often concentrated in physically demanding, difficult, and dangerous jobs. The government can alleviate the job segregation caused by the hukou system and build more connections between agricultural hukou holders’ jobs and the Internet, particularly in the era of the sharing economy. Thus, the Internet may also improve agricultural hukou holders’ job satisfaction and further improve their psychological well-being. Agricultural hukou holders are often treated unfairly in destination cities, and authorities should further perfect the channels for agricultural hukou holders to report problems to local governments. Therefore, using the Internet can provide a route for agricultural hukou holders to seek governmental support, improve their evaluation of the government, and further improve their psychological well-being. Allowing rural children who would otherwise be left behind to receive an education in cities equally is also important, and family reunions could mitigate the sleep impairment of agricultural hukou holders due to their use of the Internet and protect their psychological well-being.

This article has its theoretical contribution. With the economic development of developing countries, an increasing number of scholars have linked progress in science and technology to social inequality. Dinkelman’s research in South Africa found that electrification enabled rural residents to enter the modern labor market more, which in turn eased the employment inequality between urban and rural areas in that country [57]. A study by Rosenberg et al. in India showed that when the electricity supply was increased, instead of an increase in the number of electric lights and electric fans installed in kitchens, which are more commonly used by women, men’s preferred electrical appliances were prioritized [58]. Therefore, improving resources from the supply side alone cannot change long-term unbalanced household resource allocation patterns and can even exacerbate gender inequality. Technological advancement could be an important factor affecting welfare, but its role should be comprehensively and rationally understood. We found that the Internet, as a technological advancement, has not disrupted the long-standing unbalanced model of urban–rural resource allocation and is still more conducive to improving the psychological well-being of non-agricultural users.

This paper also has some limitations. For instance, we focused on the psychological well-being of different hukou holders; thus, future research can explore how the Internet affects users’ physical health. Our research used cross-sectional data, but personal well-being may change over time. Thus, future research can use panel data to supplement our research. With improvements in infrastructure and the increase in the Internet penetration rate, it is very important to gradually relax the hukou system, adjust the dynamic imbalance from within, and improve the return rate of Internet use and Internet use capabilities for agricultural hukou holders.

## Figures and Tables

**Table 1:** Descriptive statistics for the main variables among Chinese citizens.

VarName	Definition	Obs	Mean	SD	Min	Median	Max
Psy_well	A comprehensive index of psychological well-being consisting of 7 specific indicators; Cronbach’s α = 0.7712, overall KMO = 0.7774	30,038	80.03	16.648	0.00	83.38	100.00
Internet	Internet use: 1 = yes, 0 = no	30,130	0.53	0.499	0.00	1.00	1.00
F_soc_int	Frequency of socializing via the Internet: 0 = never, 1 = once every few months, 2 = once a month, 3 = 2–3 times a month, 4 = 1–2 times a week, 5 = 3–4 times a week, 6 = almost every day	30,124	2.65	2.815	0.00	0.00	6.00
Inform_int	Importance of the Internet for the respondent to obtain information: 1 = very unimportant…, 5 = very important	30,100	2.89	1.649	1.00	3.00	5.00
Int_aware_politic	How many days the respondent has learned about politics via the Internet in the past week	30,128	1.99	2.823	0.00	0.00	7.00
Agrihk	Current hukou status: agricultural hukou = 1, non-agricultural hukou = 0	30,131	0.74	0.439	0.00	1.00	1.00
Female	Gender: female = 1, male = 0	30,131	0.50	0.500	0.00	1.00	1.00
Age	Respondent’s age in 2018	30,131	46.70	16.876	16.00	48.00	96.00
Single	Marital status: single = 1, otherwise = 0	30,131	0.23	0.419	0.00	0.00	1.00
Education	Years of education	28,541	7.67	5.059	0.00	9.00	23.00
Incomelevel	Respondent’s rating of his or her income in the local area: 1 = lowest…, 5 = highest	28,223	2.91	1.075	1.00	3.00	5.00
Lifesatisf	Respondent’s rating of his or her life satisfaction: 1 = very dissatisfied…, 5 = very satisfied	30,124	4.01	0.959	1.00	4.00	5.00
Socialstatus	Respondent’s rating of his or her local social status: 1 = lowest…, 5 = highest	30,025	3.09	1.079	1.00	3.00	5.00
CCP	Political background: Chinese Communist Party member = 1, otherwise = 0	27,571	0.01	0.107	0.00	0.00	1.00
Smoke	Smoking in the past month: 1 = yes, 0 = no	30,130	0.29	0.453	0.00	0.00	1.00
Drinkalcohol	Drinking more than 3 times a week in the past month: 1 = yes, 0 = no	30,128	0.15	0.357	0.00	0.00	1.00
Jobsatisf	Satisfaction with work: 1 = very unsatisfied…, 5 = very satisfied	22,938	3.64	0.965	1.00	4.00	5.00
Evaluategov	Overall evaluation of the work of the county or county-level city/district government in the last year: 1 = worse than before, 2 = no achievement, 3 = not much achievement, 4 = certain achievements, 5 = great achievements	29,558	3.44	0.962	1.00	4.00	5.00
Poorsleep	Frequency of poor sleep in the past week: 1 = less than one day, 2 = 1–2 days, 3 = 3–4 days, 4 = 5–7 days	30,129	1.82	0.937	1.00	2.00	4.00
Cellphone	Use of a mobile phone: 1 = yes, 0 = no	30,131	0.92	0.267	0.00	1.00	1.00

**Table 2:** RIF (re-centered influence function) regression of different statistics for non-agricultural and agricultural hukou holders.

Variables	(1)	(2)	(3)	(4)	(5)	(6)	(7)	(8)	(9)	(10)
	urban_q10	rural_q10	urban_q25	rural_q25	urban_mean	rural_mean	urban_q75	rural_q75	urban_q90	rural_q90
	psy_well	psy_well	psy_well	psy_well	psy_well	psy_well	psy_well	psy_well	psy_well	psy_well
Internet	3.658 **	2.857 **	1.428+	0.533	1.167 *	0.346	0.199	−0.375	0.615	−0.104
	(1.240)	(0.906)	(0.846)	(0.490)	(0.519)	(0.326)	(0.478)	(0.317)	(0.456)	(0.249)
Female	−4.025 ***	−5.433 ***	−4.318 ***	−4.280 ***	−3.494 ***	−3.787 ***	−2.703 ***	−2.561 ***	−1.778 ***	−1.300 ***
	(1.149)	(0.879)	(0.784)	(0.475)	(0.481)	(0.317)	(0.443)	(0.308)	(0.423)	(0.241)
Age	0.085 *	−0.254 ***	0.103 ***	−0.111 ***	0.055 **	−0.072 ***	0.058 ***	0.000	0.055 ***	0.008
	(0.041)	(0.031)	(0.028)	(0.017)	(0.017)	(0.011)	(0.016)	(0.011)	(0.015)	(0.008)
Single	−5.872 ***	−8.963 ***	−2.194 **	−4.504 ***	−2.480 ***	−3.634 ***	−0.354	−1.234 ***	0.035	−0.591 *
	(1.202)	(0.896)	(0.819)	(0.485)	(0.503)	(0.323)	(0.463)	(0.314)	(0.442)	(0.246)
Education	1.037 ***	1.274 ***	0.492 ***	0.643 ***	0.377 ***	0.514 ***	0.117 *	0.225 ***	−0.007	0.111 ***
	(0.131)	(0.092)	(0.089)	(0.050)	(0.055)	(0.033)	(0.050)	(0.032)	(0.048)	(0.025)
Incomelevel	0.395	1.288 ***	−0.080	1.025 ***	−0.011	0.814 ***	0.139	0.523 ***	−0.015	0.212 *
	(0.549)	(0.356)	(0.374)	(0.192)	(0.230)	(0.128)	(0.212)	(0.125)	(0.202)	(0.098)
Lifesatisf	7.783 ***	6.362 ***	6.187 ***	4.712 ***	4.853 ***	4.071 ***	2.957 ***	2.763 ***	1.945 ***	1.676 ***
	(0.551)	(0.363)	(0.376)	(0.196)	(0.231)	(0.131)	(0.213)	(0.127)	(0.203)	(0.100)
Socialstatus	1.476 **	0.389	1.461 ***	0.082	1.148 ***	0.240+	0.724 ***	0.202	0.473 *	0.134
	(0.563)	(0.361)	(0.384)	(0.195)	(0.236)	(0.130)	(0.217)	(0.127)	(0.207)	(0.099)
CCP	−0.138	−1.947	0.466	−0.131	−1.430	0.084	−1.067	0.894	1.419	−1.558
	(3.928)	(4.033)	(2.679)	(2.182)	(1.645)	(1.453)	(1.514)	(1.413)	(1.445)	(1.106)
Smoke	−1.669	−1.518+	−1.536+	−0.938+	−0.758	−0.669 *	−0.454	−0.239	−0.455	0.054
	(1.272)	(0.920)	(0.868)	(0.498)	(0.533)	(0.331)	(0.491)	(0.322)	(0.468)	(0.252)
Drinkalcohol	−0.081	1.662+	0.891	0.150	0.545	0.473	0.632	0.326	−0.156	0.225
	(1.429)	(0.992)	(0.975)	(0.537)	(0.599)	(0.357)	(0.551)	(0.348)	(0.526)	(0.272)
Constant	12.679 **	33.389 ***	36.388 ***	51.655 ***	54.967 ***	61.935 ***	78.905 ***	79.327 ***	89.918 ***	89.668 ***
	(3.917)	(2.578)	(2.672)	(1.395)	(1.641)	(0.929)	(1.510)	(0.903)	(1.441)	(0.707)
Observations	5711	18,356	5711	18,356	5711	18,356	5711	18,356	5711	18,356
R-squared	0.078	0.075	0.097	0.092	0.143	0.135	0.084	0.065	0.048	0.035

Note: Standard errors are in parentheses; *** *p* < 0.001, ** *p* < 0.01, * *p* < 0.05, + *p* < 0.1; non-agricultural hukou holders are labelled “urban”, and agricultural hukou holders are labelled “rural”.

**Table 3:** RIF decomposition of differences in psychological well-being between non-agricultural and agricultural hukou holders.

Variables	(1)	(2)	(3)	(4)	(5)	(6)	(7)	(8)	(9)	(10)	(11)	(12)	(13)	(14)	(15)
	rif_q10			rif_q25			rif_mean			rif_q75			rif_q90		
	Overall	Explained	Unexplained	Overall	Explained	Unexplained	Overall	Explained	Unexplained	Overall	Explained	Unexplained	Overall	Explained	Unexplained
Internet		0.409 *	1.205		0.015	1.180+		0.006	0.940 *		−0.096	0.593		−0.027	0.606+
		(0.159)	(0.947)		(0.091)	(0.610)		(0.059)	(0.382)		(0.060)	(0.368)		(0.046)	(0.340)
Control		Yes	Yes		Yes	Yes		Yes	Yes		Yes	Yes		Yes	Yes
Group_1	62.228 ***			73.062 ***			82.353 ***			95.822 ***			100.970 ***		
	(0.474)			(0.326)			(0.206)			(0.184)			(0.172)		
Group_2	56.619 ***			69.603 ***			78.709 ***			92.310 ***			97.772 ***		
	(0.334)			(0.188)			(0.128)			(0.120)			(0.094)		
Difference	5.609 ***			3.460 ***			3.644 ***			3.512 ***			3.199 ***		
	(0.580)			(0.376)			(0.242)			(0.219)			(0.196)		
Explained	4.240 ***			2.149 ***			1.745 ***			0.879 ***			0.423 ***		
	(0.397)			(0.223)			(0.158)			(0.137)			(0.101)		
Unexplained	1.369 *			1.310 **			1.899 ***			2.633 ***			2.776 ***		
	(0.629)			(0.408)			(0.258)			(0.252)			(0.220)		
Constant			−20.002 ***			−15.379 ***			−6.965 ***			−0.291			−0.389
			(5.228)			(3.016)			(2.057)			(1.657)			(1.457)

Note: Standard errors are in parentheses; *** *p* < 0.001, ** *p* < 0.01, * *p* < 0.05, + *p* < 0.1; “group_1” represents non-agricultural hukou holders, and “group_2” represents agricultural hukou holders.

**Table 4:** Mechanism analysis (1) of the different effects of the Internet on the two categories of hukou holders.

Variables	(1)	(2)	(3)	(4)	(5)	(6)
	Urban	Rural	Urban	Rural	Urban	Rural
	psy_well	psy_well	psy_well	psy_well	psy_well	psy_well
F_soc_int	0.192 *	0.070				
	(0.089)	(0.059)				
Inform_int			−0.070	−0.387 ***		
			(0.155)	(0.093)		
Int_aware_politic					0.159 *	0.054
					(0.072)	(0.054)
Control	Yes	Yes	Yes	Yes	Yes	Yes
Constant	55.129 ***	61.899 ***	56.485 ***	63.785 ***	55.691 ***	62.152 ***
	(1.625)	(0.917)	(1.698)	(0.933)	(1.571)	(0.871)
Observations	5711	18,352	5699	18,342	5710	18,356
R-squared	0.143	0.135	0.143	0.136	0.143	0.135

Note: Standard errors are in parentheses; *** *p* < 0.001, * *p* < 0.05; non-agricultural hukou holders are labelled “urban”, and agricultural hukou holders are labelled “rural”.

**Table 5:** Mechanism analysis (2) of the different effects of the Internet on the two categories of hukou holders.

Variables	(1)	(2)	(3)	(4)	(5)	(6)	(7)	(8)	(9)	(10)	(11)	(12)
	Urban		Rural		Urban		Rural		Urban		Rural	
	psy_well	jobsatisf	psy_well	jobsatisf	psy_well	evaluategov	psy_well	evaluategov	psy_well	poorsleep	psy_well	poorsleep
Jobsatisf	1.671 ***		1.092 ***									
	(0.272)		(0.136)									
Evaluategov					0.947 ***		0.809 ***					
					(0.214)		(0.124)					
Poorsleep									−5.619 ***		−6.823 ***	
									(0.196)		(0.120)	
Internet	0.857	0.062+	0.005	−0.035+	1.458 **	0.102 **	0.122	−0.068 ***	1.792 ***	0.050	0.419	0.057 **
	(0.632)	(0.037)	(0.343)	(0.020)	(0.522)	(0.033)	(0.329)	(0.020)	(0.485)	(0.033)	(0.302)	(0.019)
Control	Yes	Yes	Yes	Yes	Yes	Yes	Yes	Yes	Yes	Yes	Yes	Yes
Constant	49.859 ***	2.228 ***	58.485 ***	2.041 ***	51.194 ***	2.246 ***	58.470 ***	2.570 ***	65.303 ***	2.075 ***	73.096 ***	1.830 ***
	(2.100)	(0.118)	(1.043)	(0.059)	(1.693)	(0.102)	(0.980)	(0.056)	(1.560)	(0.101)	(0.877)	(0.052)
Observations	3919	3919	15,486	15,486	5588	5588	18,096	18,096	5718	5718	18,387	18,387
RMSEA	0.000		0.000		0.000		0.000		0.000		0.000	
CFI	1.000		1.000		1.000		1.000		1.000		1.000	

Note: Standard errors are in parentheses; *** *p* < 0.001, ** *p* < 0.01, + *p* < 0.1; non-agricultural hukou holders are labelled “urban”, and agricultural hukou holders are labelled “rural”. RMSEA represents the root mean squared error of approximation, and CFI represents the comparative fit index.

**Table 6:** Heterogeneity analysis of Internet use among non-agricultural and agricultural hukou holders.

Variables	(1)	(2)	(3)	(4)	(5)	(6)	(7)	(8)	(9)	(10)
	Urban	Rural	Urban	Rural	Urban	Rural	Urban	Rural	Urban	Rural
	psy_well	psy_well	psy_well	psy_well	psy_well	psy_well	psy_well	psy_well	psy_well	psy_well
Internet	−0.154	−0.035	−2.863	−1.758+	0.754	0.070	3.028 **	1.332 *	1.013	−0.254
	(0.669)	(0.404)	(1.966)	(1.023)	(0.528)	(0.331)	(1.047)	(0.536)	(1.196)	(0.734)
Internet*female	2.530 **	0.775								
	(0.809)	(0.485)								
Internet*age			0.073 *	0.047 *						
			(0.034)	(0.022)						
Internet*single					4.609 ***	3.346 ***				
					(1.121)	(0.663)				
Internet*education							−0.209 *	−0.143 *		
							(0.102)	(0.062)		
Internet*incomelevel									0.055	0.207
									(0.385)	(0.227)
Control	Yes	Yes	Yes	Yes	Yes	Yes	Yes	Yes	Yes	Yes
Constant	55.985 ***	62.183 ***	57.755 ***	62.804 ***	54.132 ***	61.466 ***	54.450 ***	61.664 ***	55.045 ***	62.166 ***
	(1.672)	(0.942)	(2.100)	(1.011)	(1.651)	(0.933)	(1.660)	(0.936)	(1.730)	(0.963)
Observations	5711	18,356	5711	18,356	5711	18,356	5711	18,356	5711	18,356
R-squared	0.145	0.135	0.144	0.135	0.146	0.136	0.144	0.135	0.143	0.135

Note: Standard errors are in parentheses; *** *p* < 0.001, ** *p* < 0.01, * *p* < 0.05, + *p* < 0.1; non-agricultural hukou holders are labelled “urban”, and agricultural hukou holders are labelled “rural”.

**Table 7:** Extended regression model to address any potential concerns about endogeneity.

Variables	(1)	(2)	(3)	(4)
	erm_urban		erm_rural	
	psy_well	Internet	psy_well	Internet
Internet	3.184 *		0.644	
	(1.375)		(1.440)	
Cellphone		2.413 ***		2.211 ***
		(0.150)		(0.086)
Control	Yes	Yes	Yes	Yes
Constant	52.435 ***	−1.970 ***	60.272 ***	−2.237 ***
	(1.853)	(0.149)	(1.198)	(0.086)
Corr(e.psy_well, e.internet)	−0.073		−0.024	
	(0.056)		(0.055)	
Log likelihood	−26882.251		−89192.814	
Significance of χ^2^	<0.001		<0.001	
Observations	5718	5718	18,387	18,387

Note: Standard errors in are parentheses; *** *p* < 0.001, * *p* < 0.05, + *p* < 0.1; the prefix “e.” represents residuals; non-agricultural hukou holders are labelled “urban”, and agricultural hukou holders are labelled “rural”.

**Table 8:** Robustness test using the Tobit model.

Variables	(1)	(2)	(3)	(4)	(5)	(6)	(7)	(8)
	tobit_urban	tobit_rural	tobit_urban	tobit_rural	tobit_urban	tobit_rural	tobit_urban	tobit_trural
	psy_well	psy_well	psy_well	psy_well	psy_well	psy_well	psy_well	psy_well
Internet	1.690 **	0.001						
	(0.584)	(0.353)						
F_soc_int			0.284 **	0.028				
			(0.100)	(0.064)				
Inform_int					−0.003	−0.517 ***		
					(0.175)	(0.102)		
Int_aware_politic							0.223 **	0.078
							(0.082)	(0.059)
Control	Yes	Yes	Yes	Yes	Yes	Yes	Yes	Yes
Constant	55.271 ***	63.158 ***	55.493 ***	62.970 ***	56.979 ***	65.181 ***	56.381 ***	62.928 ***
	(1.944)	(1.098)	(1.925)	(1.086)	(2.006)	(1.108)	(1.871)	(1.042)
Observations	5712	18,356	5712	18,352	5700	18,342	5711	18,356

Note: Standard errors are in parentheses; *** *p* < 0.001, ** *p* < 0.01; non-agricultural hukou holders are labelled “urban”, and agricultural hukou holders are labelled “urban”.
